# Supplementary material for: Reconciling Mining with the Conservation of Cave Biodiversity: A Quantitative Baseline to Help Establish Conservation Priorities
Source: PLoS One. 2016 Dec 20;11(12):e0168348. doi: 10.1371/journal.pone.0168348 (PMC5173368; doi:10.1371/journal.pone.0168348)
Supplement: S1 Dataset — (ZIP) [file pone.0168348.s002.zip › Taxa/Serra Norte/SN_2013/N4WS/Lista N4WS-75.pdf]

CAVIDADE N4WS-0075

Classe	Ordem	Fam/Outros	Gên/Outros	Espécie	S-U	U-S	Zon.
Annelida	Oligochaeta			sp.		1	P
Arachnida	Acari	(Oribatida)		sp.1	X	X	P
Arachnida	Acari	(Uropodina)		sp.2	X		P
Arachnida	Acari	Argasidae	*Ornithodoros*	sp.1	X	X	E/P
Arachnida	Amblypygi	Charinidae	*Charinus*	sp.1	3	3	P
Arachnida	Amblypygi	Phrynidae	*Heterophrynus*	*longicornis*	3	6	P
Arachnida	Araneae	Corinnidae	*Creugas*	sp.1	6	4	E/P
Arachnida	Araneae	Ctenidae		jovem	2	2	E/P
Arachnida	Araneae	Ochyroceratidae	*Ochyrocera*	sp.1		X	E/P
Arachnida	Araneae	Ochyroceratidae	*Speocera*	sp.1	X		P
Arachnida	Araneae	Prodidomidae		jovem		X	E
Arachnida	Araneae	Scytodidae	*Scytodes*	*eleonorae*	1	2	E
Arachnida	Araneae	Segestriidae		jovem	X		E
Arachnida	Araneae	Theridiidae	*Achaearanea*	sp.1		X	P
Arachnida	Araneae	Theridiidae	*Anelosimus*	sp.1		X	E
Arachnida	Araneae	Theridiidae		sp.4	X	X	E/P
Arachnida	Araneae	Theridiosomatidae	*Plato*	sp.1	X	X	P
Arachnida	Pseudoscorpiones	Chernetidae		sp.1	X	X	E/P
Arachnida	Ricinulei	Ricinoididae	*Cryptocellus*	*tarsilae*		X	E/P
Arachnida	Schizomida	Hubbardiidae	aff. *Rowlandius*	sp.1	X	X	E/P
Chilopoda	Scolopendromorpha	Scolopocryptopidae	*Dinocryptops*	*miersii*	1		P
Diplopoda	Polydesmida	Chelodesmidae		sp.1		1	P
Diplopoda	Polydesmida	Pyrgodesmidae		sp.1	X	X	E/P
Diplopoda	Spirostreptida	Pseudonannolenidae	*Pseudonannolene*	sp.2	1		P
Entognatha	Collembola	Cyphoderidae		sp.1	X	X	E/P
Entognatha	Collembola	Paronellidae		sp.1		X	E
Entognatha	Collembola	Paronellidae		sp.4	X	X	P
Entognatha	Diplura	Campodeidae		sp.1	X	X	E/P
Insecta	Coleoptera	Chrysomelidae		sp.20		X	P
Insecta	Coleoptera	Elateridae		jovem		X	E
Insecta	Coleoptera	Tenebrionidae		jovem		X	E
Insecta	Diptera	Ceratopogonidae		sp.	X	X	P
Insecta	Diptera	Culicidae	*Culex*	sp.	X		E
Insecta	Diptera	Keroplatidae	*Euceroplatus*	sp.		X	
Insecta	Diptera	Psychodidae	*Sciopemyia*	*sordellii*	X	X	E
Insecta	Diptera	Sciaridae	*Bradysia*	sp.		X	E
Insecta	Heteroptera	Cydnidae	*Pangaeus*	sp.1	X	X	E/P
Insecta	Heteroptera	Lygaeidae	Rhyparochrominae	sp.1		X	E
Insecta	Heteroptera	Nabidae	Prostemmatinae	jovem		X	E
Insecta	Heteroptera	Reduviidae	Emesinae	jovem	2		E
Insecta	Heteroptera	Schizopteridae	Schizopterinae	sp.1	X	X	E
Insecta	Heteroptera	Veliidae	Veliinae	jovem		X	E
Insecta	Homoptera	Cixiidae		jovem		X	E
Insecta	Hymenoptera	Formicidae	*Camponotus*	*atriceps*		2	E/P
Insecta	Hymenoptera	Formicidae	*Camponotus*	sp.1	X		P
Insecta	Hymenoptera	Formicidae	*Crematogaster*	sp.1	X		E
Insecta	Hymenoptera	Formicidae	*Crematogaster*	sp.3		X	E
Insecta	Hymenoptera	Formicidae	*Gnamptogenys*	*striatula*	X		P
Insecta	Hymenoptera	Formicidae	*Hypoponera*	sp.1	X		E/P

Insecta	Hymenoptera	Formicidae	*Solenopsis*	sp.1	X		P
Insecta	Hymenoptera	Formicidae	*Solenopsis*	sp.2		X	P
Insecta	Hymenoptera	Formicidae	*Wasmannia*	sp.1	X		E
Insecta	Isoptera	Termitidae	*Atlantitermes*	sp.	X	X	E
Insecta	Lepidoptera	Noctuoidea		sp.1		2	
Insecta	Neuroptera	Myrmeleontidae		jovem	1		E
Insecta	Orthoptera	Phalangopsidae	*Phalangopsis*	sp.	47	245	E/P
Insecta	Thysanura	Nicoletiidae		sp.1	X	X	E/P
Gastropoda	Pulmonata	Systrophiidae	*Happia*	sp.	X	X	E/P
Malacostraca	Isopoda	Armadillidae		sp.1	X		E
Malacostraca	Isopoda	Dubioniscidae		sp.1	X	X	E/P
Symphyla		Scutigerellidae	*Hanseniella*	sp.1	X	X	E/P
Amphibia	Anura	Strabomantidae	*Pristimantis*	cf. *fenestratus*	6	12	E
Mammalia	Chiroptera	Phyllostomidae	*Anoura*	sp.	2		
Mammalia	Chiroptera	Phyllostomidae	*Carollia*	*perspicillata*	5	30	
